# Morphine-3-glucuronide upregulates PD-L1 expression *via* TLR4 and promotes the immune escape of non-small cell lung cancer

**DOI:** 10.20892/j.issn.2095-3941.2020.0442

**Published:** 2021-02-15

**Authors:** Kaiyuan Wang, Jian Wang, Ting Liu, Wenwen Yu, Nan Dong, Chen Zhang, Wenbin Xia, Feng Wei, Lili Yang, Xiubao Ren

**Affiliations:** 1 Department of Anesthesiology, Tianjin Medical University Cancer Institute and Hospital, National Clinical Research Center for Cancer, Key Laboratory of Cancer Prevention and Therapy, Tianjin, Tianjin’s Clinical Research Center for Cancer, Key Laboratory of Cancer Immunology and Biotherapy, Tianjin 300060, China; 2 Department of Immunology, Tianjin Medical University Cancer Institute and Hospital, National Clinical Research Center for Cancer, Key Laboratory of Cancer Prevention and Therapy, Tianjin, Tianjin’s Clinical Research Center for Cancer, Key Laboratory of Cancer Immunology and Biotherapy, Tianjin 300060, China; 3 Department of Cancer Biobank, Tianjin Medical University Cancer Institute and Hospital, National Clinical Research Center for Cancer, Key Laboratory of Cancer Prevention and Therapy, Tianjin, Tianjin’s Clinical Research Center for Cancer, Key Laboratory of Cancer Immunology and Biotherapy, Tianjin 300060, China; 4 Department of Biotherapy, Tianjin Medical University Cancer Institute and Hospital, National Clinical Research Center for Cancer, Key Laboratory of Cancer Prevention and Therapy, Tianjin, Tianjin’s Clinical Research Center for Cancer, Key Laboratory of Cancer Immunology and Biotherapy, Tianjin 300060, China

**Keywords:** Non-small cell lung cancer, TLR4, PD-L1, morphine-3-glucuronide, immune escape

## Abstract

**Objective:**

Patients with cancer pain are highly dependent on morphine analgesia, but studies have shown a negative correlation between morphine demand and patient outcomes. The long-term use of morphine may result in abnormally elevated serum morphine-3-glucuronide (M3G) levels. Hence, the effects of M3G on tumor progression are worth studying.

**Methods:**

The effects of M3G on PD-L1 expressions in human non-small cell lung cancer (NSCLC) cell lines were first evaluated. Activation of TLR4 downstream pathways after M3G treatment was then determined by Western blot. The effects of M3G on human cytotoxic T lymphocytes (CTL) cytotoxicity and INF-γ release was also detected. Finally, the LLC murine lung adenocarcinoma cell line were used to establish a murine lung cancer model, and the effects of M3G on tumor growth and metastasis were determined.

**Results:**

M3G promoted the expressions of PD-L1 in the A549 and H1299 cell lines in a TLR4-dependent manner (*P* < 0.05). M3G activated the PI3K and the NFκB signaling pathways, and this effect was antagonized by a TLR4 pathway inhibitor. A PI3K pathway inhibitor reversed the M3G-mediated PD-L1 upregulation. M3G inhibited the cytotoxicity of CTL on A549 cells and decreased the level of INF-γ. Repeated M3G intraperitoneal injections promoted LLC tumor growth and lung metastasis through the upregulation of tumor expressed PD-L1 and the reduction of CTL in the tumor microenvironment.

**Conclusions:**

M3G specifically activated TLR4 in NSCLC cells and upregulated PD-L1 expression through the PI3K signaling pathway, thereby inhibiting CTL cytotoxicity and finally promoting tumor immune escape.

## Introduction

Approximately 30%–50% of patients with cancer suffer from cancer-induced or cancer therapy-induced pain, of which 75%–90% could not receive satisfied relief^1^. Cancer pain can impair sleep, reduce physical and social activity, and seriously deteriorate the quality of life. Non-small cell lung cancer (NSCLC) accounts for 80%–87% of lung cancers^2^. Patients with advanced NSCLC are prone to severe cancer pain due to bone metastasis. Currently, opioids remain the primary option for the clinical treatment of cancer pain, with morphine being a representative drug. However, several retrospective studies have shown a negative correlation between the requirement for morphine and poor outcomes of NSCLC patients^3,4^. The possible mechanism may be related to the direct action of opioids on the G protein-coupled receptor (GPCR)-mu opioid receptor (MOR) expressed on peripheral immune cells and tumor cells^5–7^.

Some studies have also found that morphine can activate the NFκB pathway *via* the non-GPCRs and thus modulate tumor progression^8^. This further revealed the presence of non-classical binding sites on tumor cells that interact with morphine. Morphine-3-glucuronide (M3G) and morphine-6-glucuronide (M6G) are the active metabolites of morphine. The ratio of M3G/M6G is approximately 7.5–36. M6G binds to the classical opioid receptor, MOR, and generates a more robust and longer analgesic effect than morphine, while it also contributes to the delayed-analgesic effect of morphine^9^. However, M3G binds poorly to the MOR and antagonizes morphine analgesia. Research has shown that the clearance rates of morphine and its metabolites are remarkably reduced in patients with advanced-stage cancer, and long-term use of morphine can result in abnormally elevated levels of serum M3G^10,11^. The role of M3G in morphine-induced tumor progression is therefore worth studying.

In morphine tolerance and dependence studies, morphine was reported to stereo-selectively bind to the TLR4 in glial cells, to activate the TLR4 pathway, and to promote the release of proinflammatory cytokines^12^. M3G also binds to the TLR4/MD2 complex of glial cells and acts more strongly than morphine, whereas M6G does not bind to TLR4^13^. In tumor cells, TLR4 has been reported to be highly expressed and is associated with tumor malignancy^14,15^. Moreover, activation of TLR4 by lipopolysaccharide (LPS) can upregulate programmed death-ligand 1 (PD-L1) levels and thereby attenuate the cytotoxicity of the killer T cells (CTL) and promote the tumor immune escape^16,17^. Our previous study found that TLR4 exhibited a positive correlation with PD-L1 expression in tumor tissues of NSCLC patients receiving opioid analgesia^18^. Because M3G can activate the TLR4 pathway, it is important to determine whether M3G can regulate the PD-L1 expression through the TLR4 expressed in tumor cells, to boost tumor progression. In this study, we hypothesized that M3G specifically bound to TLR4 in NSCLC cells, to activate its downstream signaling pathways, to upregulate the expression of PD-L1, and to then attenuate the cytotoxicity of CTL, to promote tumor immune escape.

## Materials and methods

### Cell culture

Various human lung cancer cell lines including A549, H1299, H520, H460, and H446 and a murine Lewis lung carcinoma cell line, LLC1, were obtained from the American Type Culture Collection (Manassas, VA, USA). Human lung cancer cell lines were cultured in RPMI Medium 1640 (Gibco, Waltham, MA, USA) supplemented with 10% fetal bovine serum (HyClone, Logan, UT, USA). LLC cells were cultured in high glucose (4.5 g/L) Dulbecco’s Modified Eagle Medium (Gibco, Thermo Fisher Scientific) and were supplemented with 10% fetal bovine serum and 1% antibiotic-antimycotic solution (Sigma-Aldrich, St. Louis, MO, USA). The cells were then maintained in a humidified-incubator equilibrated with 5% CO_2_ at 37 °C.

### Quantitative real-time PCR (qRT-PCR)

The total RNA from cultured tumor cell lines was extracted using TRIzol reagent (Invitrogen, Carlsbad, CA, USA) following the manufacturer’s instructions. The cDNA was reverse transcribed by M-MLV Reverse Transcriptase (Promega, Madison, WI, USA). The sequences of the primers used were as follows: MOR forward: 5′-TACCGTGTGCTATGGACTGAT-3′ and MOR reverse: 5′-ATGATGACGTAAATGTGAATG-3′; TLR4 forward: 5′-GACAACCAGCCTAAAGTATT-3′ and TLR4 reverse: 5′-TGCCATTGAAAGCAACTCTG-3′; β-actin forward: 5′-TGGCACCCAGCACAATGAA-3′ and β-actin reverse: 5′-CTAAGTCATAGTCCGCCTAGAAGCA-3′. The relative quantification was performed using a real-time SYBR green fluorescence PCR kit (Takara, Shiga, Japan) using the ABI Prism 7500 Sequence Detection System (Applied Biosystems, Foster City, CA, USA) following the manufacturer’s instructions. The C_t_ values (cycle threshold) were analyzed by the ABI software and the results were further determined after calculating the comparative Ct value (2^-ΔΔCt^) by using β-actin as the internal control.

### Flow cytometry

Single cell suspensions were prepared directly from the cultured A549 and H1299 lung cancer cell lines or LLC tumor tissues and were then stained with the following conjugated, human specific monoclonal antibodies (BioLegend, San Diego, USA): PE-PD-L2, PerCP/Cy5.5-Gal-9, PE/Cy7-CD200, and PE/Cy7-PD-L1, and mouse monoclonal antibodies including PE/Cy7-PD-L1, PE/CD8, FITC/CD4, PE/Cy7-TIM3, and APC-IFN-γ (BioLegend). The appropriate isotype controls purchased from BioLegend served as the negative controls. The cells were incubated with the indicated antibodies for 30 min at 4 °C when protected from light, washed twice with phosphate-buffered saline (PBS), and then resuspended in a final volume of 300 μL of 4% paraformaldehyde solution for analysis. All samples were analyzed in 3 independent experiments, and at least 20,000 events were acquired in each test.

### Western blot

After removing the culture medium, cancer cells were washed twice with ice-cold PBS. The harvested cells were then lysed in a lysis buffer containing protease inhibitor and a phosphatase inhibitor cocktail (Roche Mannheim, Mannheim, Germany) for 30 min at 4 °C and centrifuged at 12,000 rpm for 15 min. The supernatants were collected and the protein concentrations were measured using a Pierce BCA Protein Assay Kit (Thermo Fischer Scientific, Waltham, MA, USA) according to the manufacturer’s instructions. Equal quantities of total proteins were separated on a 10% SDS-PAGE gel and transferred onto polyvinylidene difluoride (PVDF) membranes (Millipore, Burlington, MA, USA). The membranes were blocked in 5% nonfat milk for 1 h and then incubated overnight with primary antibody including rabbit anti-MOR antibody (1:1,000; Abcam, Cambridge, MA, USA), rabbit anti-TLR4 antibody (1:500; Abcam), rabbit anti-PD-L1 antibody (1:500; Abcam), and rabbit antibodies to p-ERK, ERK, p-JNK, JNK, p-p38, p38, p-Akt, Akt, p-P65, and P65 (1:1,000; Cell Signaling Technology, Danvers, MA, USA). The PVDF membranes were then incubated with horseradish peroxidase (HRP)-conjugated secondary antibodies for 1 h. The blots were developed using the ECL substrate (Amersham, Aldermaston, UK). Glyceraldehye 3-phosphate dehydrogenase (1:2,000; Abcam) served as the endogenous control for the protein quantification.

### The siRNA transfection

A total of 3 siRNAs targeting TLR4 were obtained from MilliporeSigma (Burlington, MA, USA) and the siRNA that exerted the strongest knockdown efficacy was selected for subsequent experiments. A549 cancer cells were seeded at 2 × 10^5^ cells/well in 24-well plates. After 24 h incubation at 37 °C, when A549 cells had adhered to the plastic, the cells were transfected with control siRNA or TLR4 targeting siRNA using the Lipofectamine™ RNAiMAX (Invitrogen, Carlsbad, CA, USA) protocol. After 16 h incubation at 37 °C, the A549 cells were harvested and analyzed by Western blot.

### ELISA

The A549 and H1299 cells were seeded into 24-well plates at a density of 5 × 10^5^ cells/well with 0.5 mL of the RPMI 1640 medium supplemented with 10% FBS. After ensuring attachment for 12 h, the cancer cells were treated with M3G (10 μM) or LPS (0.1 μg/mL) for 24, 48, and 72 h. The cell culture medium was collected at different time intervals and centrifuged at 1,200 rpm for 5 min. The supernatant was then aspirated for analysis. The human IL-6, TNF-α, and IL-1β ELISA kits were obtained from R&D Systems (Minneapolis, MN, USA) and the tests were performed according to the manufacturer’s instructions. The absorbance was measured at 450 nm using a microplate reader. A standard curve was drawn and the sample concentrations were calculated from the formula.

### Cell viability assay

A total of 5,000 A549, H1299, or LLC cells were seeded in each well of a 96-well microplate with 100 μL of the cell culture medium. Different concentrations of the vehicle or M3G were added to the wells and co-incubated for 24, 48, and 72 h in a CO_2_ incubator. The Cell Counting Kit-8 (CCK-8) reagent (Dojindo, Kumamoto, Japan) was added (10 μL) into each well and further incubated for 1–2 h. The absorbance was measured at 450 nm using a microplate reader. Each experiment was conducted three times.

### Patients and healthy volunteers

This study was approved by the Ethical Committee of the Tianjin Medical University Cancer Institute and Hospital (TMUCIH) (Approval No. Ek2017028) and informed consent was obtained from each participant, in accordance with the Declaration of Helsinki. Fresh tumor tissues were obtained from 6 lung cancer patients receiving pulmonary resections at the Department of Lung Carcinoma, TMUCIH. All patients had a pathology-based diagnosis of NSCLC before surgery and none of the patients received radiotherapy, chemotherapy, or other medical intervention before sample collection. Ten healthy male volunteers (ages > 18 years) were also recruited. Their blood samples were obtained for human leukocyte antigen, subtype A (HLA-A) detection and further culture of immune cells.

### HLA-A typing measurement

The HLA-A low concentration typing kit (101.401.48/78Y; Olerup SSP, Stockholm, Sweden) was used to detect the HLA-A subtype of the A549 and H1299 cell lines and the blood samples of volunteers. All experiments were performed according to the manufacturer’s instructions. The results from the agarose gel electrophoresis were analyzed according to the interpretation tables in the typing kit.

### CTL culture and target-killing experiments

Peripheral venous blood was obtained from eligible healthy volunteers. The peripheral blood mononuclear cells were collected by Ficoll sorting and the dendritic cells (DCs) and the T cells were cultured separately. The culture medium was supplemented with recombinant human granulocyte-macrophage colony-stimulating factor (R&D Systems) and IL-4 (R&D Systems) to stimulate DC maturation. Human IL-2, IL-7, and IL-15 (all from R&D Systems) were added to the cell culture media of the T cells. The expressions of DC surface-activation markers (CD80, CD83, CD14, CD11C, and HLA-DR) were determined by flow cytometry on day 10 of the DC culture. The activated mature DCs were incubated with the cultured autologous T cells. The HLA-restricted polypeptide synthesized according to the results of the HLA-A subtype screening was further used in the DC and T cell culture system to activate DC and T cells through cell antigen presentation. The CTL clones were observed microscopically after 4 days of co-incubation. The increased expression level of the surface activation marker, CD107a, was used to confirm the successful activation of CTLs.

The living cells were separated and collected by Ficoll sorting of the CTLs, and their concentration was adjusted to 2 × 10^6^/mL. These cells served as the effector cells (E). In addition, the A549 cell lines preloaded with the antigenic peptide (after treatment with M3G) were collected at a concentration of 5 × 10^4^/mL and were used as the target cells (T). The E and T cells were added to a 96-well, U-shaped plate (200 μL/well) with E/T ratios of 40:1, 20:1, and 10:1. Three replicate wells were established for each group. Each group included a natural-release group, a volume-correction control, a culture blank control, and a target-cell maximum-release group. The cells were centrifuged at 250 × *g* for 4 min and then incubated at 37 °C in a 5% CO_2_ incubator for 4 h. At 30 min before the end of the culture, 20 μL of NP40 lysate was added to each well of the target-cell maximum-release group and the volume-correction control group, and centrifuged at 250 × *g* for 4 min. After an incubation for 30 min, 50 μL of the supernatant from each well was aspirated and transferred into another 96-well plate; 50 μL of the substrate solution was then added to each well. The plate was incubated for 30 min in the dark at room temperature, 50 μL of the stop solution was added, and the absorbance was measured at 490 nm with a microplate reader. CTL cytotoxicity was calculated according to the following formula: CTL activity (%) = (experimental group – effector cell natural release group – target cell natural release group + culture medium blank control group)/(target cell maximum release group – volume correction control group – target cell natural release group + culture medium blank control group) × 100%.

### ELISpot assay for the release of IFN-γ

An ELISpot plate (Dakewe, Shenzhen, China) was precoated with human IFN-γ. After treatment with different concentrations of M3G, the cultured CTL cells (E) and the A549 cells (T) preloaded with antigenic peptides were added to the wells of the plate with E/T ratios of 40:1, 20:1, and 10:1 and cultured for 24 h. Biotin-labeled antibodies and enzyme-labeled avidin were used for chemical enzymatic visualization to observe locally circular spots formed on the membranes. The number of spots indicated the quantity of positive cells. The percentage of positive cells was calculated by dividing the number of positive cells by the total number of cells initially added to the well.

### Immunochemistry staining

For 3,3-diaminobenzidine (DAB) immunohistochemistry (IHC) staining of TLR4 and MOR, 60 paraffin-embedded human NSCLC samples were baked for 1 h at 70 °C and deparaffinized. The tissue was then fixed with formaldehyde and blocked with 5% serum for 1 h, and antigens were retrieved by heat treatment in citrate buffer (10 mM, pH 6). Endogenous peroxidase activity was inhibited using 0.3% hydrogen peroxide for 20 min. The tissues were then incubated with primary antibodies including mouse anti-TLR4 antibody (1:100; Abcam) and rabbit anti-MOR antibody (1:100; Abcam) overnight at 4 °C. After rinsing with PBS, an HRP-conjugated goat anti-mouse or goat anti-rabbit secondary antibody was added for 30 min at 37 °C. The slides were washed and incubated with DAB chromogen substrate (ZSGB-Bio, Beijing, China). The nucleus was stained by hematoxylin (Sigma-Aldrich, St. Louis, MO, USA). Finally, the tissue sections were covered with a glass coverslip and observed using light microscopy.

Tumor sections were evaluated using consensus viewpoints by 2 pathologists who were blinded to the identities of the tissues. Positive samples of TLR4 and MOR were defined as those showing membranous or cytoplasmic staining of tumor cells. Briefly, the IHC score was determined using a semi-quantitative method involving the staining intensity and the percentage of stained cells. The staining intensity was scored into 4 categories according to the color of immune reaction: negative, 0; light brown, 1; brown, 2; and dark brown, 3. The percentage of positively-stained cells was determined and scored as: ≤ 5%, 0; 6%–25%, 1; 26%–50%, 2; 51%–75%, 3; and 76%–100%, 4. The expression levels of TLR4 and MOR were obtained by multiplying the intensity and proportion scores.

For immunofluorescence (IF) staining, A549 cells with indicated treatments in exponential growth phase were fixed with 4% paraformaldehyde (PFA) for 30 min. For mice tumor samples, mice were deeply anesthetized with isoflurane and perfused intracardially with PBS. LLC tumors were removed from the mice and post-fixed in 4% PFA overnight. The samples were then dehydrated with 30% sucrose solution, embedded in Tissue-Tek O.C.T., and sliced into 8 μm sections using a cryostat. The A549 cells or tumor sections were then blocked with 0.5% bovine serum albumin in PBS for 30 min and then preincubated with primary antibodies including anti-NFκB P65 (1:500; Abcam) or anti-PD-L1 (1:500; Abcam) overnight at 4 °C. After washing 3 times with ice-cold PBS, the cells were then incubated with tetramethylrhodamine-isothiocyanate (TRITC) or cy3-conjugated goat anti-rabbit IgG antibody at room temperature, and the nuclei were stained with 4′,6-diamidino-2-phenylindole (1:1,000; Thermo Fisher Scientific). The cells were then washed twice with PBS and the images were visualized using a 1024 confocal laser microscope (Bio-Rad, Hercules, CA, USA).

### Animal experiments

All the animal experiments were approved by the Animal Ethical and Welfare Committee of TMUCIH (No. AE2014039). C57 BL/6 mice with ages of 6–8 weeks were used in the study. The murine cell line, LLC1, was lightly digested using 0.05% trypsin, followed by centrifugation to remove poorly digested cell clusters. The cells were then resuspended in PBS at a concentration of 2 × 10^6^ cells/mL. A total of 2 × 10^5^ cells in 100 μL PBS were subcutaneously injected into the right flank of mice to establish tumor models. On day 5, the mice were injected intraperitoneally (i.p.) with vehicle, M3G (10 mg/kg), TAK-242 [3 mg/kg (resatorvid); a small-molecule-specific inhibitor of Toll-like receptor (TLR) 4 signaling], or LY294002 (5 mg/kg; a PI3K pathway inhibitor) for 14 days. The tumor volume was measured every 2 or 3 days and recorded. The mice were sacrificed on day 21, and the local LLC tumor tissues and lungs were collected and photographed for further analyses.

### Statistical analysis

Prism 6.0 software (GraphPad Software, San Diego, CA, USA) was used to perform all statistical analyses and to draw the figures. The Shapiro-Wilk test was used to assess the normality of the data. Data are presented as the mean and standard error of the mean (SEM), and were compared using two-tailed Student’s *t*-tests, one-way analysis of variance, or two-way analysis of variance with repeated measurements, followed by Bonferroni’s multiple comparisons test. A two-tailed *P* value < 0.05 was considered statistically significant.

## Results

### Higher expression of TLR4 and lower expression of MOR in NSCLC cells

**Figure 1:**
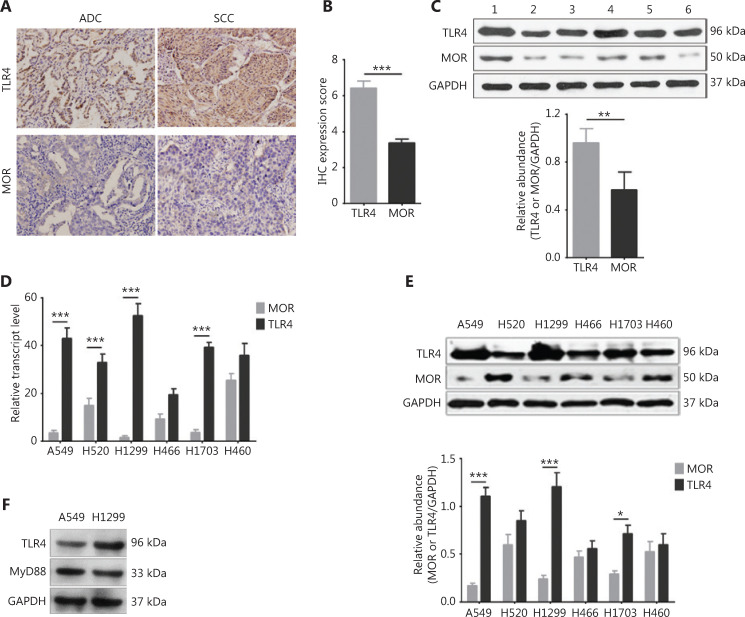
Expression of toll-like receptor 4 (TLR4) and (GPCR)-mu opioid receptor (MOR) in human lung cancer cell lines. (A) Representative images of immunohistochemcial staining (40×) of TLR4 and MOR in human adenocarcinoma (ADC) and squamous cell carcinoma (SCC) tumor tissues. (B) Quantification for A. *n* = 60 tumor samples. (C) Western blot analysis showing high expression of TRL4 and low expression of MOR in fresh human non-small cell lung cancer (NSCLC) tumor samples. Upper panel: representative Western blot bands; lower panel: quantification of the blot. *n* = 6 tumor samples. (D) The mRNA expression of TLR4 and MOR in human lung cancer cell lines including A549, H1299, H520, H1703, H446, and H460. *n* = 3 cultures. (E) Western blot analysis showing the increased expression of TLR4 and decreased expression of MOR in NSCLC cell lines. Upper panel: representative blot of TLR4 and MOR expressions; lower panel: quantification for the blot, *n* = 3 cultures. (F) MyD88 protein is expressed in the A549 and H1299 cell lines as seen from the Western blot results. Data indicate the mean ± SEM. *P < 0.05; **P < 0.01; ***P < 0.001, two-tailed Student’s *t*-test (B, C); repeated-measures two-way analysis of variance with Bonferroni’s post hoc test (D, E).

Previously, we showed that TLR4 was highly expressed in tumor tissue of NSCLC patients receiving opioid analgesia, and was correlated with poor outcomes^18^. In the present study, we compared the expression levels of TLR4 and the classic opioid receptor, MOR, in NSCLC tumor tissues. We found that the IHC expression scores of TLR4 in lung adenocarcinomas (ADCs) and squamous carcinomas (SCCs) were significantly higher than that of MOR (**Figure 1A** and **1B**, *P* < 0.001). Western blot using fresh tumor tissue from NSCLC patients also demonstrated the high level of TLR4, but not of MOR (**Figure 1C**, *P* < 0.01).

We further checked the expression levels of TLR4 and MOR in human lung cancer cell lines with different pathologies including A549 (ADC), H1299 (ADC), H520 (SCC), H1703 (SCC), H446 (small cell lung cancer), and H460 (large cell lung cancer). RT-qPCR showed that all the ADC and SCC cell lines had lower MOR expressions and higher TLR4 expressions at the mRNA level (**Figure 1D**, *P* < 0.001). The Western blot also showed similar expression trends, with A549 and H1299 possessing the highest TLR4 levels and lowest MOR levels (**Figure 1E**, *P* < 0.001). Furthermore, the myeloid differentiation primary response 88 (MyD88) protein and the adaptor protein of the TLR4 signaling pathway were also expressed in these two screened cell lines (**Figure 1F**). Based on these results, we selected the H1299 cell line for the next functional experiments.

### M3G increases the expression of PD-L1 in the A549 and H1299 cell lines in a dose-dependent manner

**Figure 2:**
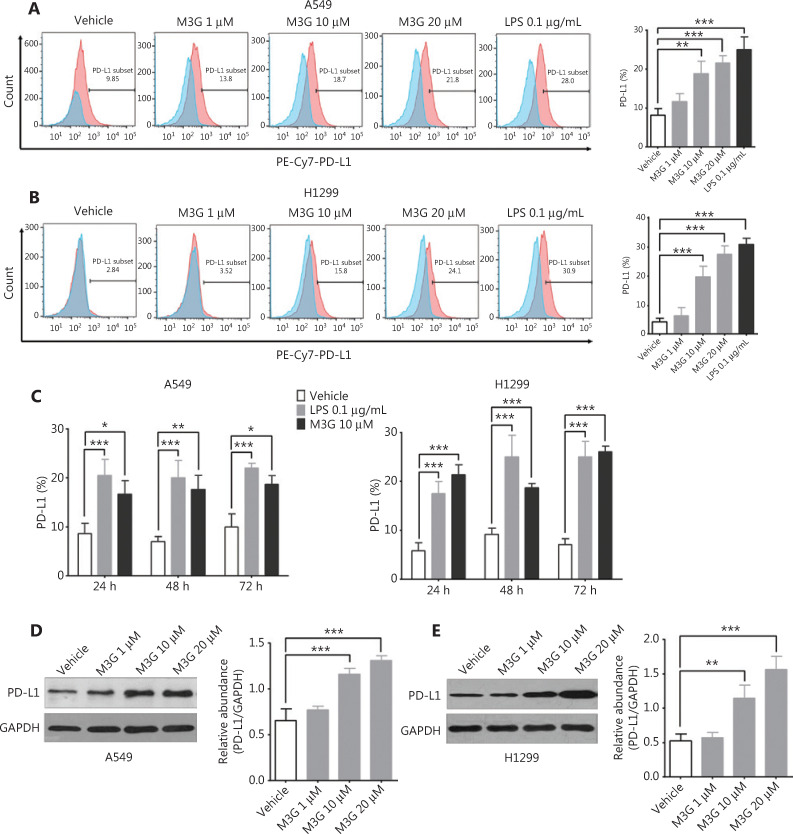
Dose-dependent upregulation of programmed death-ligand 1 (PD-L1) by morphine-3-glucuronide (M3G) in A549 and H1299 cell lines. (A) Flow cytometric analysis showing that M3G treatment for 24 h increases the membrane expression of PD-L1 in A549 cell lines in a dose-dependent manner. Left panel: representative images of flow cytometric analysis; right panel: quantification of the left panel, *n* = 3 cultures. (B) M3G treatment promotes the expression of PD-L1 in H1299 cell lines in a dose-dependent manner. Left panel: representative flow cytometry analysis images; right panel: quantification of the left panel, *n* = 3 cultures. (C) Co-incubation with 10 μM M3G or 0.1 μg/mL lipopolysaccharide for 24, 48, and 72 h upregulates the membrane expression of PD-L1 in the A549 and H1299 cell lines, *n* = 3 cultures. (D and E) Western blot analysis showing that M3G increases the total protein expression of PD-L1 in a dose-dependent manner in the A549 (D) and H1299 (E) cell lines, *n* = 3 cultures, respectively. *P < 0.05; **P < 0.01; ***P < 0.001. Data indicate the mean ± SEM. *P < 0.05; **P < 0.01; ***P < 0.001, one-way analysis of variance with Bonferroni’s post hoc test (A, B, D, E); repeated-measures two-way analysis of variance with Bonferroni’s *post hoc* test (C).

We then determined the effects of M3G on PD-L1 expressions in the A549 and H1299 cell lines. Because the serum concentration of M3G in cancer patients receiving morphine was approximately 1.6–62.1 μM^19^, we used a comparable concentration of M3G ranging from 1 μM to 20 μM in the current study. Flow cytometric analysis showed that M3G treatment for 24 h promoted the membrane expressions of PD-L1 in the A549 and H1299 cell lines in a dose-dependent manner. A significant difference was observed at 10 μM and 20 μM concentrations of M3G, as well as at 0.1 μg/mL LPS, when compared with vehicle (**Figures 2A** and **2B**, *P* < 0.01). LPS was used as a positive control because it has been reported to enhance tumor PD-L1 expression *via* TLR4^16^. We also showed that administration of either 10 μM M3G or 0.1 μg/mL LPS for 24, 48, and 72 h increased the expressions of PD-L1 in both A549 and H1299 cell lines (**Figure 2C**, *P* < 0.05). In addition, Western blot analysis showed that M3G increased the whole protein expression of PD-L1 in the A549 and H1299 cell lines in a dose-dependent manner (**Figures 2D** and **2E**, *P* < 0.01).

**Figure 3:**
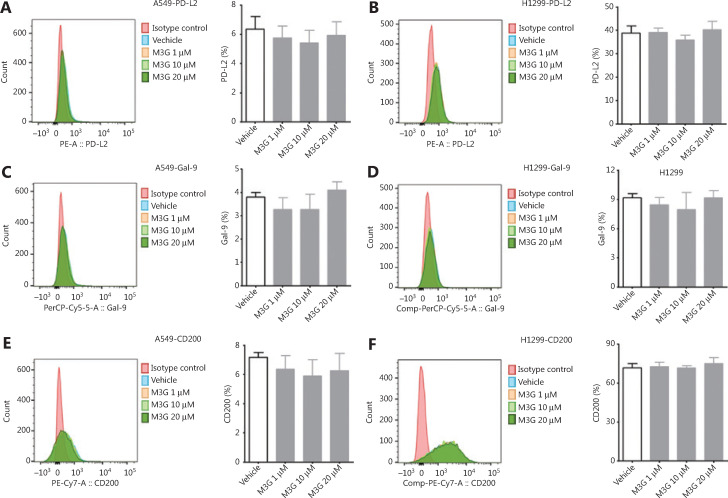
The effect of morphine-3-glucuronide (M3G) on the expression of PD-L2, Gal-9, and CD200. (A and B) Treatment with M3G (1 μM, 10 μM, and 20 μM) for 24 h does not alter PD-L2 expression in A549 (A) and H1299 cell lines (B), *n* = 3 cultures. (C–D) M3G fails to change the membrane expression level of Gal-9 in A549 (C) or H1299 cells (D), *n* = 3 cultures. (E–F) No statistical difference is observed in the expression of CD200 between vehicle treatment or treatment with different concentrations of M3G in A549 (E) and H1299 cell lines (F), *n* = 3 cultures. Data indicate the mean ± SEM, one-way analysis of variance with Bonferroni’s post hoc test.

PD-L2, Gal-9, and CD200 are other commonly recognized checkpoints that compromise the function of CTLs in the tumor microenvironment. We next determined whether M3G could modulate the expression patterns of these proteins. The results from flow cytometry showed that treatment with 1 μM, 10 μM, and 20 μM concentrations of M3G for 24 h did not change the PD-L2 expressions in both the A549 and H1299 cell lines (**Figures 3A** and **5B**, *P* > 0.05). Additionally, M3G ranging in concentrations from 1 μM to 20 μM failed to alter the membrane expressions of Gal-9 in both the A549 and H1299 cells (**Figures 3C** and **3D**, *P* > 0.05). Moreover, there was no statistical difference in the expressions of CD200 in the A549 and H1299 cell lines treated with the vehicle and those treated with different concentrations of M3G (**Figures 3E** and **3F**, *P* > 0.05). Together, the results showed that M3G treatment specifically promoted PD-L1 expression without affecting the levels of other checkpoints, including PD-L2, Gal-9, and CD200 in the A549 and H1299 cell lines.

### M3G promotes the expression of PD-L1 *via* TLR4

**Figure 4:**
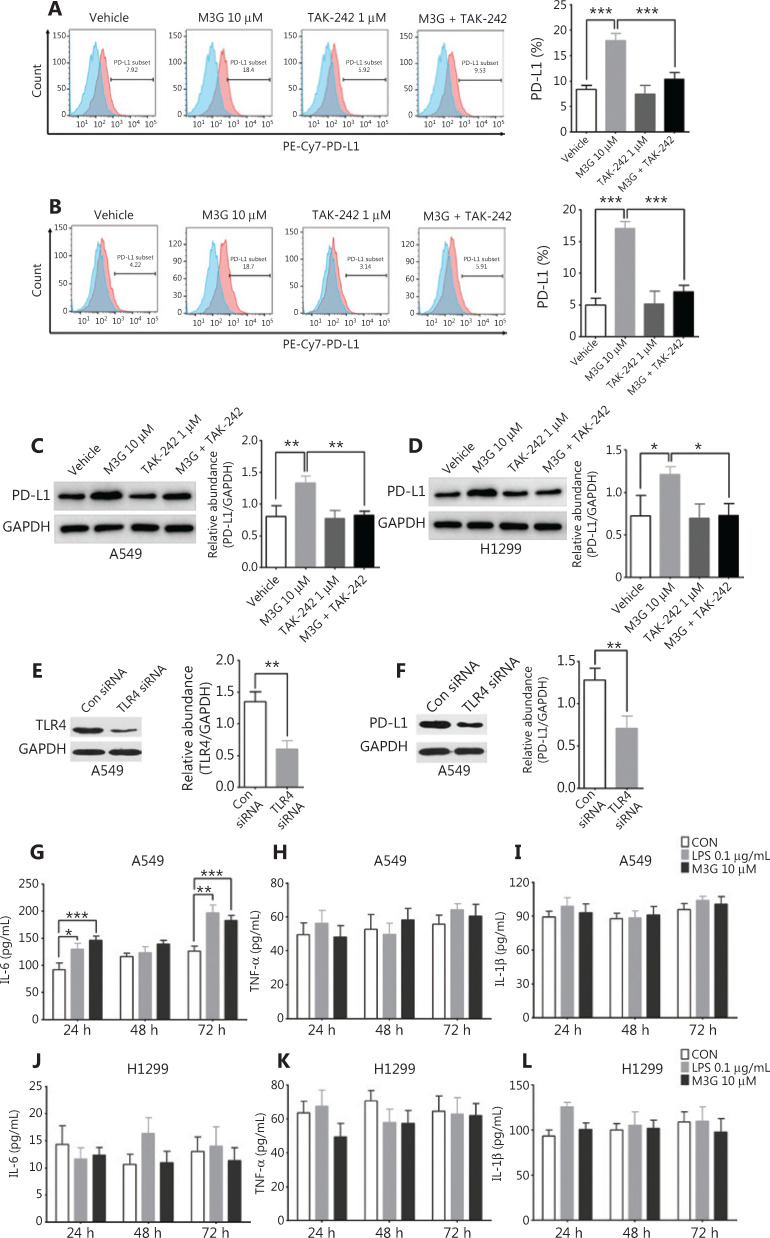
M3G (morphine-3-glucuronide) upregulates PD-L1 expression through the TLR4 pathway. (A and B) Flow cytometric analysis indicating that M3G (10 μM) promotes PD-L1 expression in A549 cells (A) and H1299 cells (B) and that this process is reversed by combined treatment with TAK-242 (1 μM) and M3G, *n* = 3 cultures, respectively. (C and D) Western blot analyses showing that the M3G-mediated increase in the level of PD-L1 protein is abolished by TAK-242 treatment (1 μM) in A549 (C) and H1299 (D) cell lines, *n* = 3 cultures, respectively. (E) TLR4 siRNA can effectively knockdown the expression of TLR4 in A549 cells, *n* = 3 cultures. (F) Western blot analysis showing that TLR4 knockdown by siRNA inhibits the upregulation of PD-L1 caused by M3G;* n* = 3 cultures. (G) Co-incubation of the cells with M3G (10 μM) or LPS (0.1 μg/mL) for 24 and 72 h significantly increases the IL-6 levels in the culture medium of the A549 cells, *n* = 3 cultures. (H and I) Treatment with M3G (10 μM) or lipopolysaccharide (0.1 μg/mL) does not affect the secretion of TNF-α (H) and IL-1β (I) in A549 cells, *n* = 3 cultures, respectively. (J–L) Treatment with M3G (10 μM) or LPS (0.1 μg/mL) for 24 h, 48 h and 72 h does not change the concentration of IL-6 (J), TNF-α (K), and IL-1β (L) in the culture medium of the H1299 cells. Data are presented as the mean ± SEM. *P < 0.05; **P < 0.01; ***P < 0.001, one-way analysis of variance with Bonferroni’s post hoc test (A–D); two-tailed Student’s *t*-test (E, F); repeated-measures two-way analysis of variance with Bonferroni’s post hoc test (G–L).

Because M3G directly bound to TRL4 in glial cells, and the activation of TLR4 in tumor cells modulated PD-L1 expression, we further examined whether the upregulation of PD-L1 in A549 and H1299 cells by M3G was mediated by TLR4. To this end, the TLR4-pathway inhibitor TAK-242 was used to inhibit TLR4 downstream signaling. Both flow cytometric analysis and Western blot showed that TAK-242 (1 μM) alone did not change the expression of PD-L1. However, co-administration of TAK-242 (1 μM) and M3G (10 μM) reversed the increased expression of PD-L1 caused by M3G (10 μM) in both the A549 and H1299 cells (**Figures 4A–D**, *P* < 0.05). We also used TLR4 siRNA to knockdown the expression of TLR4 in A549 cells, and Western blot analysis indicated that the M3G-mediated increase of the PD-L1 protein could be abolished by TLR4 knockdown (**Figures 4E** and **4F**, *P* < 0.05).

TLR4 activation may also lead to the secretion of cytokines like IL-6, TNF-α, and IL-1β. To test this hypothesis, we examined changes in the levels of the proinflammatory cytokines after M3G treatment. As seen in **Figure 4G**, co-incubation of M3G (10 μM) or LPS (0.1 μg/mL) for 24 and 72 h significantly increased the IL-6 levels in the culture media of the A549 cells (*P* < 0.05). However, there was no effect on the secretion of TNF-α and IL-1β after M3G (10 μM) or LPS (0.1 μg/mL) treatment of A549 cells (**Figure 4H** and **4I**, P > 0.05). For H1299 cells, treatment with M3G (10 μM) or LPS (0.1 μg/mL) for 24, 48, and 72 h did not change the concentrations of IL-6, TNF-α, and IL-1β (**Figure 4J**, **4K** and **4L**, all *P* > 0.05).

### M3G activates the PI3K and NFκB signaling pathways in the A549 cell line

**Figure 5:**
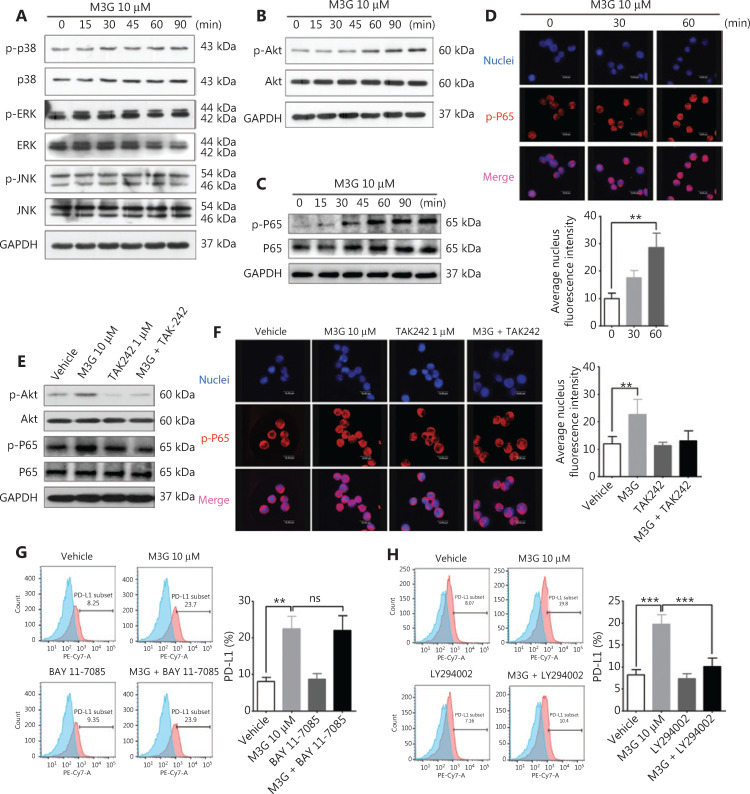
PI3K pathway mediates morphine-3-glucuronide (M3G)-induced upregulation of PD-L1 *via* TLR4. (A) The effect of M3G (10 μM) treatment on the expressions of phosphorylated and whole P38, ERK, and JNK in A549 cells at the indicated time points. (B) M3G increases the level of Akt phosphorylation in a time-dependent manner without changing the levels of total Akt protein in A549 cells. (C) M3G treatment enhances the expression of phosphorylated P65 in whole cell lysates. (D) Immunofluorescence staining showing increased p-P65 nuclear localization after M3G (10 μM) treatment. Upper panel: representative staining images. Lower panel: quantification for p-P65 nuclear staining intensity, *n* = 3 cultures. (E) Combined treatment with TAK-242 and M3G reverses M3G-mediated increases in the phosphorylation levels of Akt and P65 in A549 cells. (F) The change of p-P65 nuclear localization after M3G (10 μM) or M3G combined with TAK-242 treatment using immunofluorescence staining. Left panel: representative images. Right panel: quantification for the left panel, *n* = 3 cultures. (G) Flow cytometric analysis showing that M3G promotes the expression of PD-L1, and that the combination of BAY 11-7085 and M3G fails to change PD-L1 levels. Left panel: representative flow cytometry analysis images; right panel: quantification for left panel, *n* = 3 cultures. (H) LY294002 significantly reverses the M3G (10 μM)-mediated upregulation of PD-L1. Left panel: representative flow cytometry analysis images; right panel: quantification of the left panel, *n* = 3 cultures. Data are presented as mean ± SEM. **P < 0.01; ***P < 0.001, one-way ANOVA with Bonferroni’s post hoc test.

To observe the activation of the downstream signaling pathways after M3G treatment, 10 μM M3G was added to the A549 cell cultures for 0, 15, 30, 45, 60, and 90 min. The cells were collected at the above time points and lysed so that the phosphorylation of key proteins in the MAPK, PI3K, and NFκB signaling pathways involved in the TLR4 activation could be detected by the Western blot. As shown in **Figure 5A**, M3G did not alter the levels of phosphorylated and whole proteins of P38, ERK, and JNK, indicating that M3G did not activate the MAPK signaling pathway. However, treatment with 10 μM M3G increased the level of phosphorylated Akt (p-Akt) without changing the level of the whole Akt protein in a time-dependent manner (**Figure 5B**), showing that M3G activated the Akt-PI3K signaling pathway. M3G also enhanced the expression of phosphorylated P65 (p-P65) in whole cell lysates, when determined by Western blot (**Figure 5C**). Immunofluorescence staining showed that the p-P65 levels in tumor nuclei increased after M3G treatment for 30 and 60 min (**Figure 5D**). Taken together, the results showed that the NFκB signaling pathway was activated after treatment with M3G.

Next, we examined whether M3G-induced activation of PI3K and NFκB signaling pathways was TLR4-dependent. A549 cells were co-cultured with 10 μM M3G or 10 μM M3G, together with 1 μM TAK-242, and 1 μM TAK-242 alone for 60 min. The cells were then collected to determine the phosphorylated levels of Akt and P65 using Western blot. The results showed that M3G treatment increased the phosphorylations of both Akt and P65. However, in the A549 cell lysate the combination of TAK-242 with M3G reversed the increases of p-Akt and p-P65 without changing their whole protein levels (**Figure 5E**). Immunofluorescence staining further verified that co-incubation with TAK-242 suppressed the effect of M3G, including p-P65 nuclear localization in A549 cells (**Figure 5F**). Together, these results showed that M3G activated the PI3K and NFκB signaling pathways *via* TLR4.

### The PI3K pathway modulates the M3G-induced upregulation of PD-L1

Based on the screened PI3K and NFκB signaling pathways, we further investigated which pathway led to the increased expressions of PD-L1 after M3G treatment. The PI3K pathway inhibitor, LY294002, or the NFκB pathway inhibitor, BAY 11-7085, were used alone or in combination with 10 μM M3G for the treatment of A549 cell cultures for 24 h. Flow cytometric analysis showed that independent treatment with either LY294002 or BAY 11-7085 failed to change the levels of PD-L1 in A549 cells. However, when combined with M3G, LY294002 significantly blocked the upregulation of PD-L1 (**Figure 5G**, *P* < 0.001), while BAY 11-7085 did not change the expression of PD-L1 when compared to that when M3G alone was used as the treatment (**Figure 5H**, *P* > 0.05). The results showed that enhanced expression of PD-L1 after interaction of M3G with TLR4 was mediated through the PI3K pathway instead of the NFκB pathway.

### M3G impaired the cytotoxicity and INF-γ release of human CTL in CTL target-killing experiments

**Figure 6:**
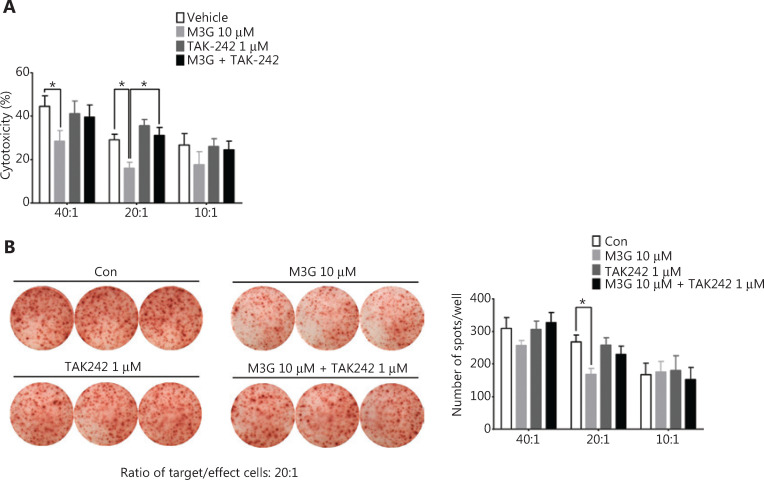
Morphine-3-glucuronide (M3G) undermines the function of CTL. (A) M3G significantly reduces the cytotoxicity of CTL in the CTL target-killing experiments towards the A549 cells at the ratios of 40:1 and 20:1 (CTL/A549) and this effect is reversed by TAK-242 treatment, *n* = 3 independent experiments. (B) The ELISpot experiment shows that A549 cells pretreated with 10 μM M3G for 24 h decreases the release of INF-γ from the cytotoxic T lymphocytes (CTLs) in the co-culture of CTLs and A549 at a ratio of 20:1. Left panel: representative images for ELISpot; right panel: quantification for the left panel, *n* = 3 independent experiments. Data are presented as the mean ± SEM. *P < 0.05, one-way analysis of variance with Bonferroni’s post hoc test.

To test the effect of M3G on T cell function and tumor immune escape, we performed *in vitro* CTL target-killing experiments. First, the PCR and agarose gel electrophoresis analyses were used to detect the HLA-A subtype of the A549 and H1299 cells. The results showed that the HLA-A subtypes of the A549 cell line were HLA-A25 and HLA-A30, while the subtypes of the H1299 cell line were HLA-A24 and HLA-A32 (**Supplementary Figure S1**). Next, the blood samples from healthy volunteers were analyzed for the HLA-A subtype. The samples with HLA-A30 showed a successful match to the A549 cell line and hence were used for culturing, and were further stimulated into mature CTLs. Accordingly, the A549 cells were selected as the target cells. The HLA-A30 specific peptide, IMYNYPAML, was predicted according to the dataset of the Immune Epitope Database and Analysis Resource (https://www.iedb.org/) and was synthesized. The A549 cells were co-cultured with 10 μM M3G, 1 μM TAK-242, and 10 μM M3G + 1 μM TAK-242 in the presence of the IMYNYPAML peptide for 24 h. The cells were then counted and cultured together with mature CTLs (effector cells) in distinct ratios of effector/target cells (40:1, 20:1, and 10:1) for 6 h. The results showed that M3G significantly reduced the cytotoxicity of CTLs in the killing of target cells (A549 cells) when used in the ratios of 40:1 and 20:1 (effector/target cells, *P* < 0.05); this effect was further reversed by the addition of TAK-242 (**Figure 6A**, *P* < 0.05). In addition, M3G (1 μM, 10 μM, and 20 μM) alone did not affect the viability of A549 and H1299 cells (**Supplementary Figure S2**, *P* > 0.05).

IFN-γ is a key moderator of CTL-mediated anti-tumor immunity^20^. We therefore determined the effect of M3G on the release of IFN-γ from CTLs. The ELISpot experiment showed that pretreatment of A549 cells with 10 μM M3G for 24 h significantly reduced the release of INF-γ from the CTL in co-cultures of CTL and A549 cells at a ratio of 20:1 (**Figure 6B**, right panel, *P* < 0.05). Together, these results indicated that M3G reduced CTL-mediated cytotoxicity through the TLR4 pathways in the A549 cells, and thus impaired immune surveillance.

### M3G promotes the LLC tumor growth and lung metastasis through the upregulation of tumor expressed PD-L1 *in vivo*

**Figure 7:**
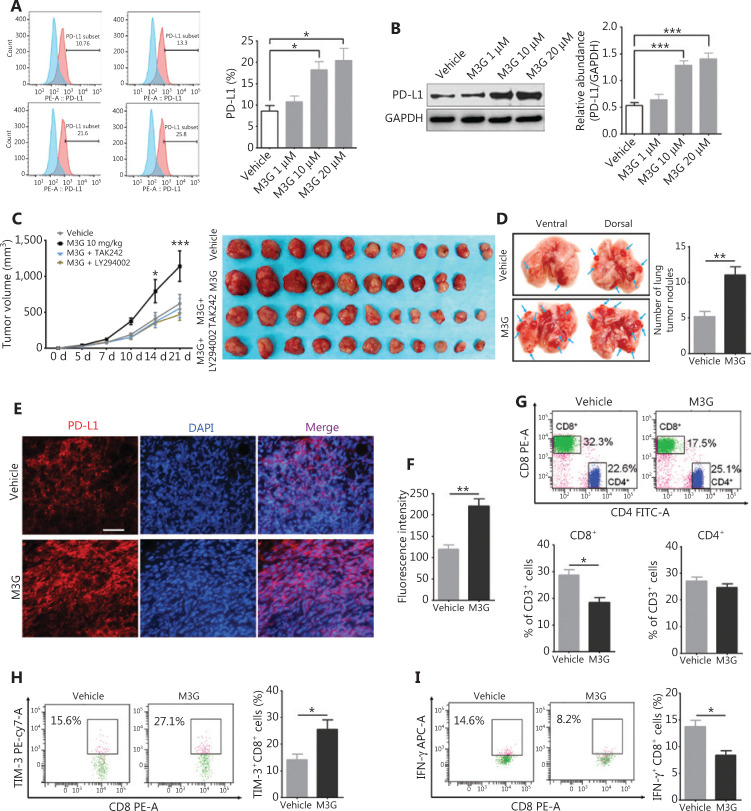
Morphine-3-glucuronide (M3G) promotes LLC tumor growth and lung metastasis *via* the upregulation of PD-L1 *in vivo*. (A) Flow cytometry showing increased expression of PD-L1 after M3G treatment at the indicated concentration in LLC cells;* n* = 3 cultures. (B) Changes of PD-L1 protein levels after M3G treatment (1 μM–20 μM) in LLC lysates from Western blot. *n* = 3 cultures. (C) Repeated intraperitoneal injections of M3G (10 mg/kg) for 14 days increases local tumor volume while this could be further reversed by combined therapy with TAK-242 or LY294002 on day 17 and day 21 after LLC subcutaneous inoculation;* n* = 10–11 mice. Left panel: time-dependent tumor growth curve. Right panel: representative photographs of tumors extracted from mice with the indicated treatment on day 21 of the experiment. (D) Number of tumor nodules in lungs from mice treated with vehicle or M3G on day 21 after LLC inoculation. Left panel: representative images of lung metastasis. Right panel: quantification for the left panel. *n* = 8 mice. (E–F) Immunofluorescences staining showing PD-L1 expression in tumors from mice treated with M3G or vehicle on day 10 after LLC inoculation. (E) Representative immunostaining images, bar, 50 μM. (F) Quantification for E;* n* = 5 mice. (G) Changes of CD4^+^ T cells and CD8^+^ T cells in tumor tissues from mice treated with M3G or vehicle. Upper panel: representative flow cytometry image. Lower panel: quantification for proportion of CD4^+^ T cells and CD8^+^ T cells;* n* = 5 mice. (H) Flow cytometry showing change of TIM-3 expression after M3G treatment in CD8^+^ T cells;* n* = 4 mice. (I) Change of IFN-γ in CD8^+^ cells after M3G treatment in tumor tissues. Left panel: representative flow cytometry image. Right panel: quantification for the upper panel;* n* = 4 mice. Data indicate the mean ± SEM. *P < 0.05; **P < 0.01; ***P < 0.001, one-way analysis of variance with Bonferroni’s post hoc test (A, B); repeated-measures two-way analysis of variance with Bonferroni’s post hoc test (C); two-tailed Student’s *t*-test (D, F, G–I).

Finally, we verified the above findings in immune intact preclinical animal models. In accordance with results in A549 and H1299 cell lines, TLR4 was highly expressed in murine lung ADC cell line-LLC cells and M3G treatment failed to regulate their viability (**Supplementary Figure S3**). However, M3G treatment promoted both membrane expression of PD-L1 (**Figure 7A**, *P* < 0.05) and the whole protein expression level of PD-L1 (**Figure 7B**, *P* < 0.001) in LLC. In C57 BL/6 mice subcutaneously inoculated with LLC, daily i.p. injections of M3G (10 mg/kg) for 2 weeks significantly boosted tumor growth compared to the vehicle group, and this effect was reversed by combined treatment with TAK242 or LY294002 (**Figure 7C**, left panel, *P* < 0.05). On day 21, the mice were sacrificed, and the lungs were collected. We found mice treated with M3G exhibited more lung tumor nodules compared to mice treated with vehicle (**Figure 7D**, *P* < 0.01). Thus, repeated M3G application could not only promote the local tumor progression, but also led to increased lung metastasis.

**Figure 8:**
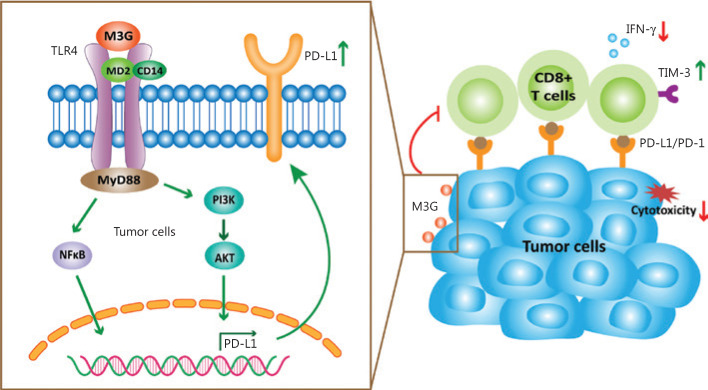
Schematic model of morphine-3-glucuronide (M3G) promoting non-small cell lung cancer (NSCLC) immune escape *via* toll-like receptor 4 (TLR4) and programmed death ligand 1 (PD-L1). The combination of M3G and TLR4 activates the PI3K pathway and induces the upregulation of PD-L1 in NSCLC cells. This will further increase the expression level of TIM-3, reduce the release of IFN-γ, and damage the cytotoxicity of CD8^+^ T cells, leading to immune escape of the NSCLC cells.

On day 10 after LLC inoculation, the tumor samples were harvested for PD-L1 staining and the analysis of immune cells. We found that there was a dramatic increase of tumor expressed PD-L1 in the M3G group compared to the vehicle group (**Figure 7E** and **7F**, *P* < 0.01). Flow cytometry analysis on tumor tissues revealed a significant reduction of the number of CD8^+^ T cells in the M3G-treated mice, but not in vehicle-treated mice (**Figure 7G**, *P* < 0.05). There was no statistical difference in the number of CD4^+^ T cells between the two groups (**Figure 7G**, *P* > 0.05). Moreover, we detected the expression levels of TIM-3 and IFN-γ as exhaustion and activation markers of CD8^+^ T cells, respectively. We found that M3G treatment increased the expression of TIM-3 but reduced the level of IFN-γ on CD8^+^ T cells in tumor tissues (**Figure 7H** and **7I**, *P* < 0.05). Taken together, these results indicated that M3G upregulated tumor PD-L1 expression and decreased CTLs in the tumor microenvironment, to facilitate tumor immune escape (**Figure 8**).

## Discussion

Lung cancer is one of the most common malignant tumors worldwide, and the incidence of its associated pain ranks third among all cancers^21^. Previous studies have shown that although the severity of cancer-related pain is inversely related to the survival of the patients with advanced NSCLC, the control of pain through opioids does not improve their outcomes. Moreover, morphine application is an independent risk factor for the poor prognosis of NSCLC patients^4^. For patients with early stage NSCLC undergoing radical lung cancer surgery, the 5-year recurrence increased while the overall survival decreased in patients with higher intra-operative and postoperative morphine demands. For patients with advanced NSCLC who cannot receive surgical therapy, the amount of opioid usage is also negatively correlated with their outcomes^3,4^. Therefore, it is of great importance to clarify the mechanisms and the relationship between morphine treatment and the progression of NSCLC.

The previous view was that morphine aggravates tumor growth mainly through the effect of immune suppression and the promotion of tumor angiogenesis. First, morphine analgesia can provoke the immunosuppressive glucocorticoid production through the hypothalamic-pituitary-adrenal (HPA) axis, thereby compromising immune functions^22^. Second, MOR is expressed in various immune cells such as in multinucleated lymphocytes, macrophages, and T lymphocytes. Morphine treatment can weaken the chemotaxis and phagocytosis of macrophages, and damage the proliferation and antibody formation of B cells *via* MOR^7^. Moreover, the action of morphine on specific tumor expressed classical GPCRs (μ, κ, and δ opioid receptors) can activate the MAPK/ERK or Akt/MTOR signaling pathways, and thus can simultaneously transactivate vascular endothelial growth factor to induce tumor angiogenesis and promote tumor cell proliferation^23^.

In this study, we first investigated the role of the primary morphine metabolite, M3G, in tumor progression by studying its interaction with tumor-expressed TLR4. TLR4 belongs to the family of pattern recognition receptors and is involved in immune regulation and the inflammatory response^24^. The activation of the TLR4 signaling pathway in tumor cells by LPS can induce tumor proliferation through the COX-2 and EGFR pathways, and can promote metastasis *via* the NADPH oxidase 1-dependent ROS pathway^14,25^. In the present study, we showed that M3G also acted on NSCLC cells expressing TLR4, which led to TLR4 activation. Moreover, M3G-mediated TLR4 activation further upregulated PD-L1 expression and promoted the secretion of IL-6. As a result, M3G reduced CTL-mediated cytotoxicity and the escape of tumors from the immune system. This is consistent with recent studies in which the stimulation of TLRs, especially TLR4, increased the expression of PD-L1 in tumor cells, independent of INF-γ. In acute myeloid leukemia, TLR4 agonists induce PD-L1 expression and inhibit the killing function of the CTLs, which can be blocked by the MEK signaling pathway inhibitors^17^. PD-L1 expression on the plasma cells of patients with multiple myeloma is upregulated by INF-γ or the TLR4 ligand, and in a MYD88/TRAF6- or the MEK-dependent manner^26^. Activation of TLR4 in bladder cancer cell lines also increases the expression of PD-L1 by activating the ERK and JNK signaling pathways^16^. In the present study, we further showed that the activation of TLR4 by M3G did not affect the levels of other common immune checkpoints, including PD-L2, Gal-9, and CD200 on the tumor cells, but just modulated PD-L1 expression.

As an important mechanism for negatively regulating T cell activation to prevent autoimmune responses under physiological conditions, PD-L1 is dynamically expressed in tumor cells and promotes their escape from immune surveillance^27^. Generally, the regulation of PD-L1 expression is closely related to oncogenic activation of tumor pro-survival signaling pathways^28,29^. For example, activation of the MAPK pathway drives the expression of PD-L1 in melanoma cells. Similarly, the MAPK activation in NSCLC also upregulates the PD-L1 expression through the activation of EGFR^30^. The PI3K/Akt pathway may modulate the PD-L1 expression in a cell- and tissue-type dependent manner *via* transcriptional or post-transcriptional mechanisms. Increased levels of transcription factors such as NF κB, hypoxia-inducible factor alpha (HIF-1α), and STAT3 have also been associated with increased PD-L1 expression, and result in the attenuation of T cell function^31–34^. In this study, we found that M3G treatment activated the PI3K and NFκB signaling pathways in A549 cells, and that this effect was antagonized by the TLR4 pathway blocker, TAK-242. We further showed that PI3K signaling pathway blockers, and not NFκB signaling pathway blockers, specifically reversed the upregulation of PD-L1 induced by M3G. Thus, mechanistically, the PI3K signaling pathway mediates the M3G induced PD-L1 upregulation *via* TLR4.

In the present study, we provided 2 lines of evidence to functionally verify the role of M3G in NSCLC immune escape. First, in CTL target-killing experiments, M3G pretreatment on human NSCLC cells remarkably reduced the cytotoxicity of CTLs and the release of INF-γ. This demonstrated that modulation of M3G on tumor PD-L1 expression could affect the amount and function of human CTLs. Second, in NSCLC preclinical animal models, repeated M3G systemic applications accelerated tumor growth and lung metastasis. Further analysis confirmed that this effect was mediated by increased levels of PD-L1 and reduction of CTLs in the tumor microenvironment. These 2 observations also enhanced the clinical relevance and significance of our findings. Because the PD-1/PD-L1 axis has been recently reported to be involved in the modulation of acute and chronic pain including bone cancer pain^35–38^, it will be important to determine the possible effect of M3G-induced PD-L1 upregulation during the development of cancer pain. Whether the upregulation of PD-L1 caused by M3G increases the response of anti-PD-1 or anti-PD-L1 treatment should also be determined in future studies.

## Conclusions

Overall, the current study showed that M3G activated TLR4 in NSCLC cells and upregulated PD-L1 expression through the PI3K signaling pathway, thereby inhibiting CTL cytotoxicity and finally promoting tumor escape from the immune system.
