# Supplementary material for: Morphine-3-glucuronide upregulates PD-L1 expression via TLR4 and promotes the immune escape of non-small cell lung cancer
Source: Cancer Biol Med. 2021 Feb 15;18(1):155–71. doi: 10.20892/j.issn.2095-3941.2020.0442 (PMC7877184; doi:10.20892/j.issn.2095-3941.2020.0442)
Supplement: Supplementary file 1 [file cbm-18-155-s001.pdf]

Supplementary materials

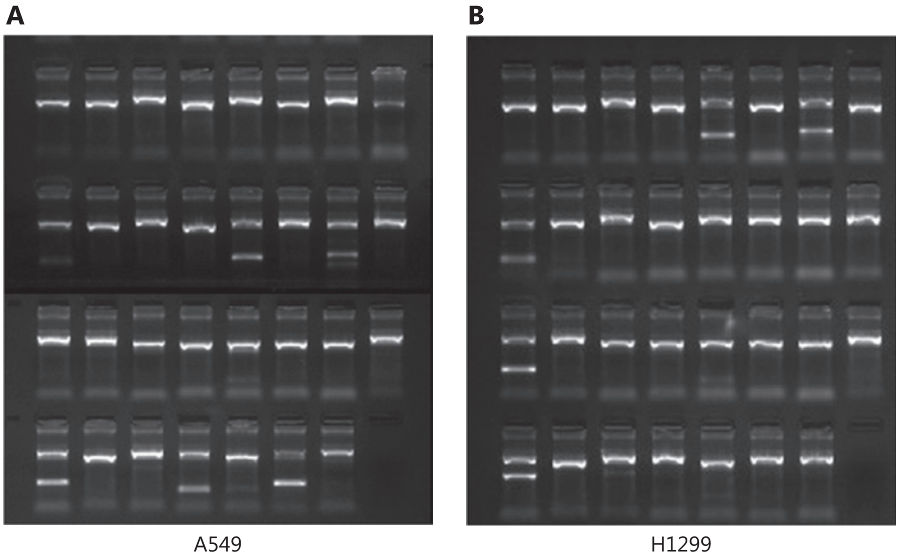

Figure S1 Agarose gel electrophoresis for the HLA-A subtype of H1299 and A549 cell line. (A–B) Agarose gel electrophoresis showing the HLA-A subtype of the A549 cell line (A) and the HLA-A subtype of H1299 cells (B).

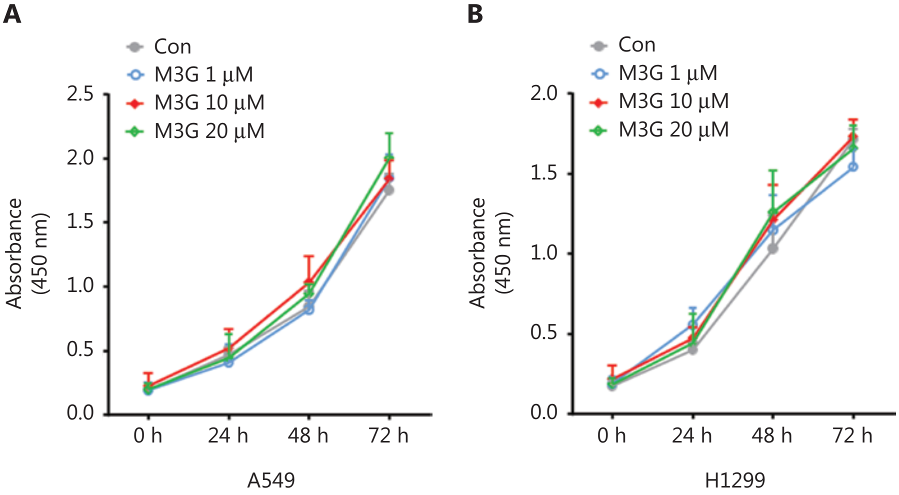

Figure S2 Morphine-3-glucuronide **(**M3G) does not change the viabilities of A549 and H1299 cells. (A–B) Treatment with increasing concentrations of M3G (1 μM, 10 μM, and 20 μM) does not affect the viability of A549 cells (A) and H1299 cells (B) after 24, 48, and 72 h of co-culture. Data indicate the mean ± SEM. Repeated-measures two-way analysis of variance with Bonferroni's post hoc test.

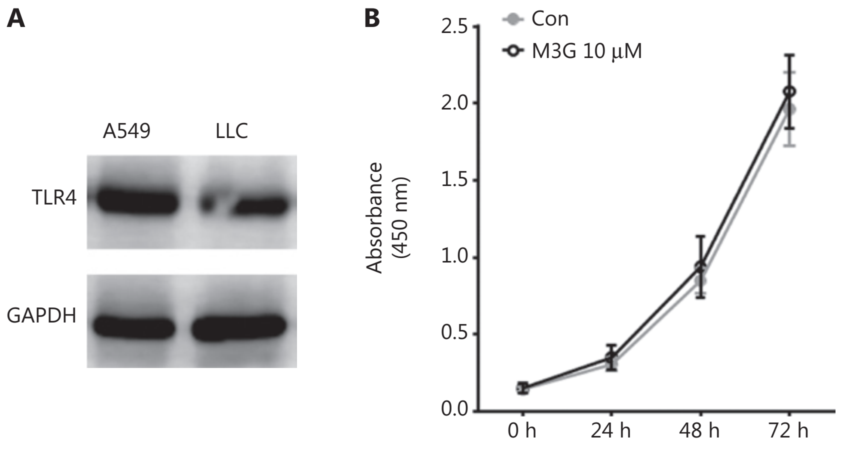

Figure S3 High expression of toll-like receptor 4 (TLR4) in LLC cells. (A) Western blot showing the high expression of TLR4 in LLC and A549 cells. (B) Morphine-3-glucuronide (10 μM) did not affect the viability of LLC cells after 24, 48, and 72 h of co-culture using CCK8 tests; *n* = 3 cultures. Data indicate the mean ± SEM. Repeated-measures two-way analysis of variance with Bonferroni's post hoc test.
